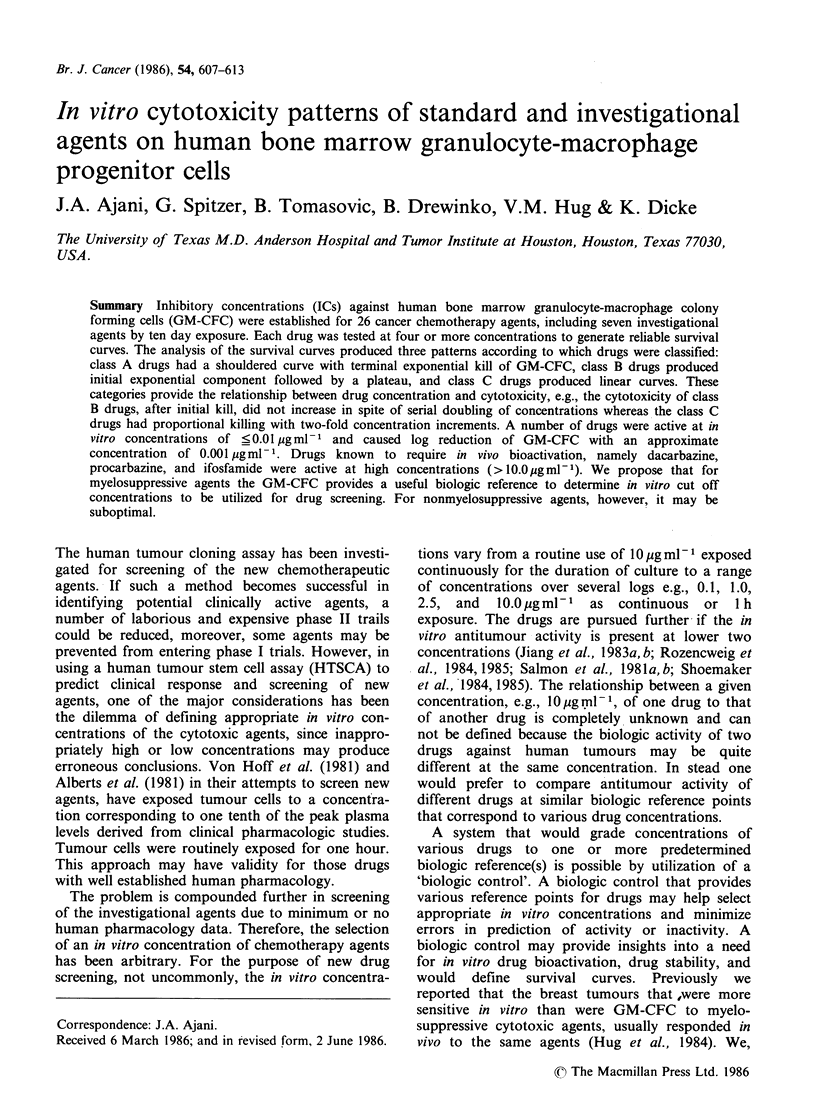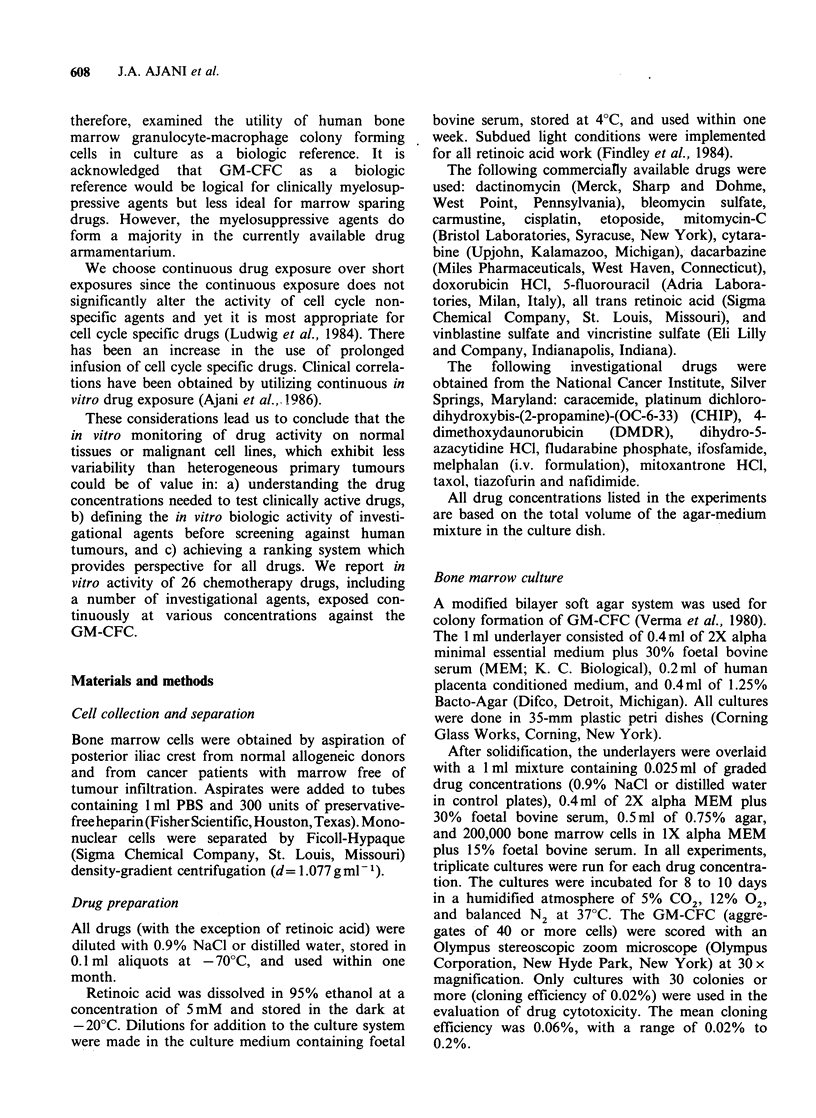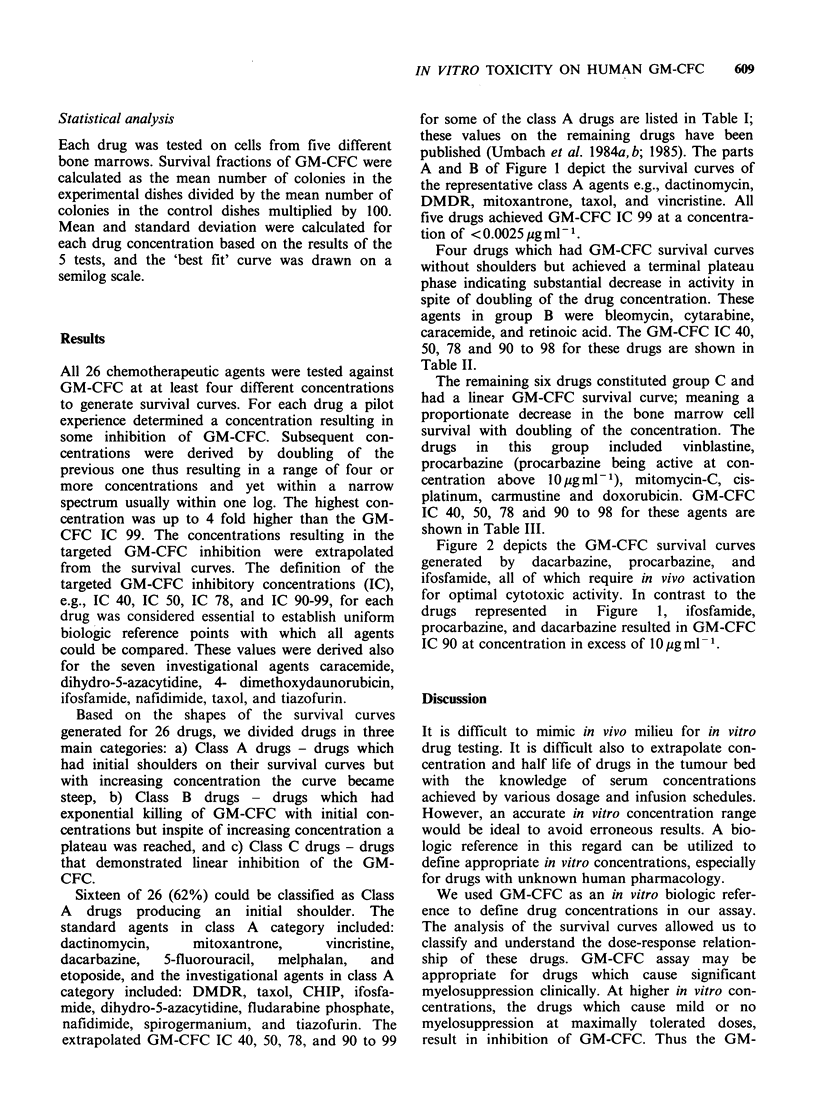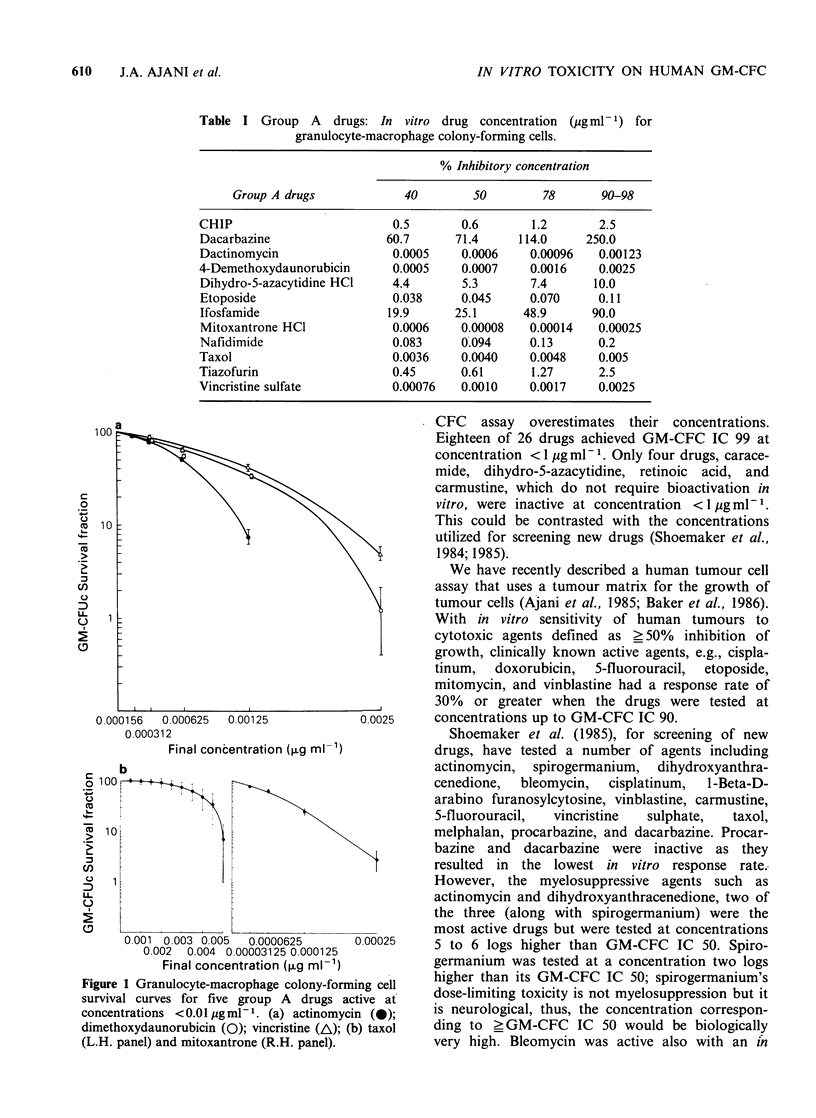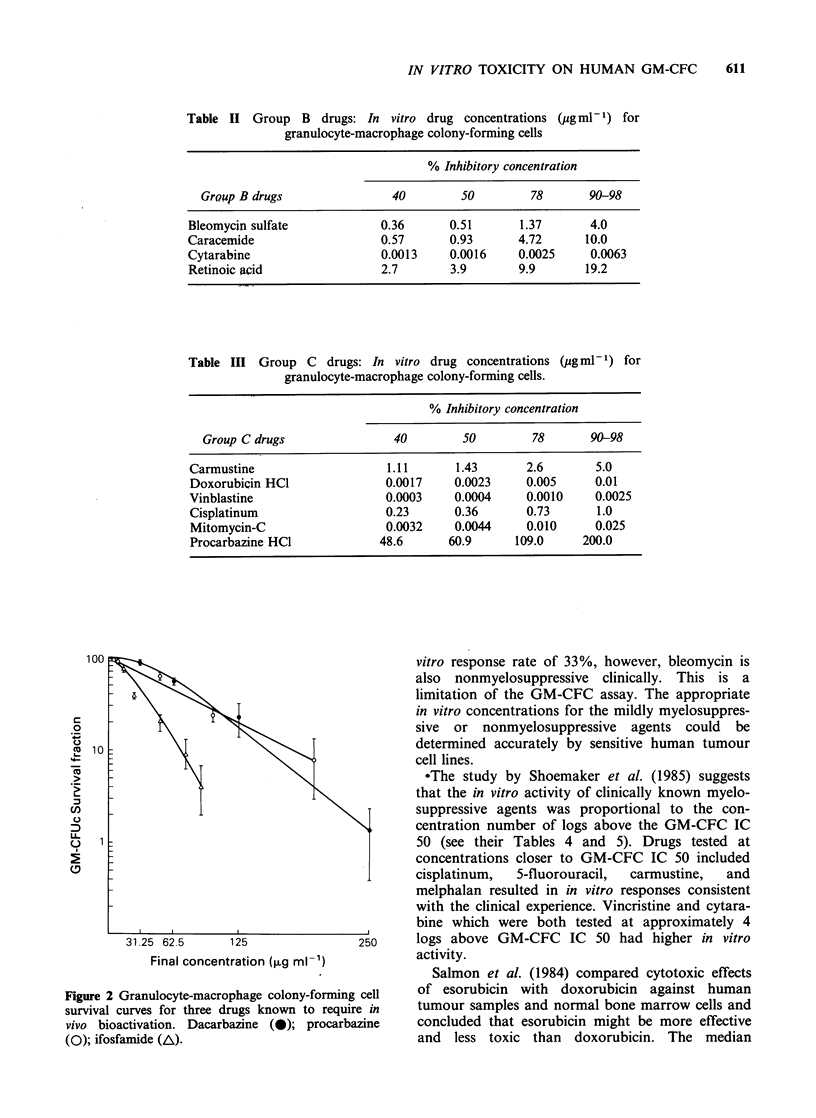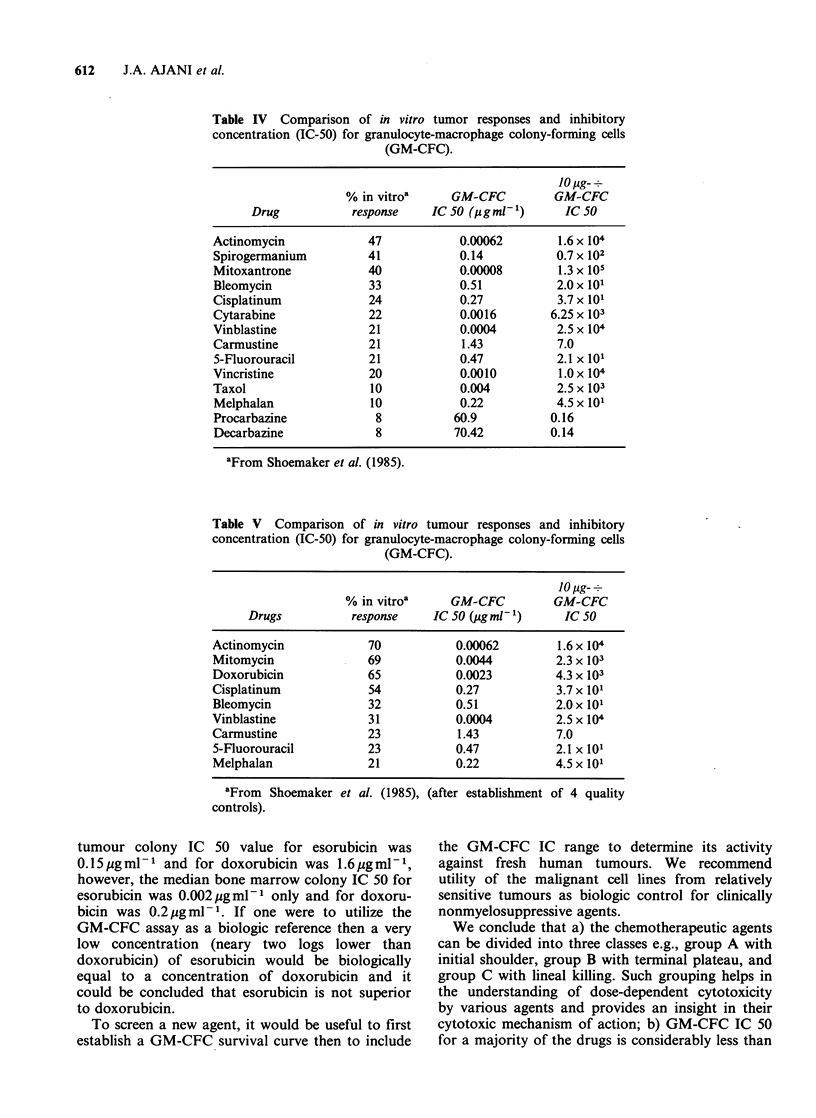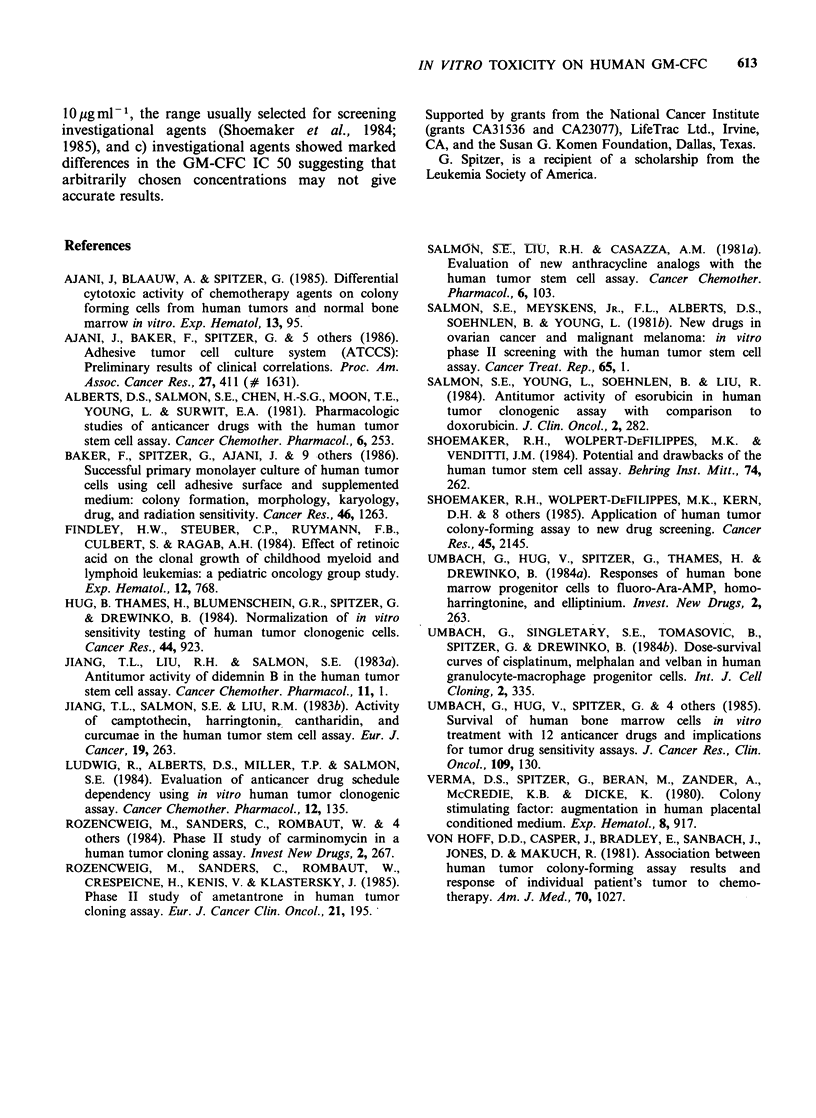# In vitro cytotoxicity patterns of standard and investigational agents on human bone marrow granulocyte-macrophage progenitor cells.

**DOI:** 10.1038/bjc.1986.216

**Published:** 1986-10

**Authors:** J. A. Ajani, G. Spitzer, B. Tomasovic, B. Drewinko, V. M. Hug, K. Dicke

## Abstract

Inhibitory concentrations (ICs) against human bone marrow granulocyte-macrophage colony forming cells (GM-CFC) were established for 26 cancer chemotherapy agents, including seven investigational agents by ten day exposure. Each drug was tested at four or more concentrations to generate reliable survival curves. The analysis of the survival curves produced three patterns according to which drugs were classified: class A drugs had a shouldered curve with terminal exponential kill of GM-CFC, class B drugs produced initial exponential component followed by a plateau, and class C drugs produced linear curves. These categories provide the relationship between drug concentration and cytotoxicity, e.g., the cytotoxicity of class B drugs, after initial kill, did not increase in spite of serial doubling of concentrations whereas the class C drugs had proportional killing with two-fold concentration increment. A number of drugs were active at in vitro concentrations of less than or equal to 0.01 microgram ml-1 and caused log reduction of GM-CFC with an approximate concentration of 0.0001 microgram ml-1. Drugs known to require in vivo bioactivation, namely dacarbazine, procarbazine, and ifosfamide were active at high concentrations (greater than 10.0 micrograms ml-1). We propose that for myelosuppressive agents the GM-CFC provides a useful biologic reference to determine in vitro cut off concentrations to be utilized for drug screening. For nonmyelosuppressive agents, however, it may be suboptimal.


Br. J. Cancer (1986), **54**, 607–613

### *In vitro* cytotoxicity patterns of standard and investigational agents on human bone marrow granulocyte-macrophage progenitor cells

J.A. Ajani, G. Spitzer, B. Tomasovic, B. Drewinko, V.M. Hug & K. Dicke

*The University of Texas M.D. Anderson Hospital and Tumor Institute at Houston, Houston, Texas 77030, USA.*

**Summary** Inhibitory concentrations (ICs) against human bone marrow granulocyte-macrophage colony forming cells (GM-CFC) were established for 26 cancer chemotherapy agents, including seven investigational agents by ten day exposure. Each drug was tested at four or more concentrations to generate reliable survival curves. The analysis of the survival curves produced three patterns according to which drugs were classified: class A drugs had a shouldered curve with terminal exponential kill of GM-CFC, class B drugs produced initial exponential component followed by a plateau, and class C drugs produced linear curves. These categories provide the relationship between drug concentration and cytotoxicity, e.g., the cytotoxicity of class B drugs, after initial kill, did not increase in spite of serial doubling of concentrations whereas the class C drugs had proportional killing with two-fold concentration increments. A number of drugs were active at *in vitro* concentrations of $\leqq 0.01\,\mu g\,ml^{-1}$ and caused log reduction of GM-CFC with an approximate concentration of $0.001\,\mu g\,ml^{-1}$. Drugs known to require *in vivo* bioactivation, namely dacarbazine, procarbazine, and ifosfamide were active at high concentrations ($>10.0\,\mu g\,ml^{-1}$). We propose that for myelosuppressive agents the GM-CFC provides a useful biologic reference to determine *in vitro* cut off concentrations to be utilized for drug screening. For nonmyelosuppressive agents, however, it may be suboptimal.

The human tumour cloning assay has been investigated for screening of the new chemotherapeutic agents. If such a method becomes successful in identifying potential clinically active agents, a number of laborious and expensive phase II trails could be reduced, moreover, some agents may be prevented from entering phase I trials. However, in using a human tumour stem cell assay (HTSCA) to predict clinical response and screening of new agents, one of the major considerations has been the dilemma of defining appropriate *in vitro* concentrations of the cytotoxic agents, since inappropriately high or low concentrations may produce erroneous conclusions. Von Hoff *et al.* (1981) and Alberts *et al.* (1981) in their attempts to screen new agents, have exposed tumour cells to a concentration corresponding to one tenth of the peak plasma levels derived from clinical pharmacologic studies. Tumour cells were routinely exposed for one hour. This approach may have validity for those drugs with well established human pharmacology.

The problem is compounded further in screening of the investigational agents due to minimum or no human pharmacology data. Therefore, the selection of an *in vitro* concentration of chemotherapy agents has been arbitrary. For the purpose of new drug screening, not uncommonly, the *in vitro* concentrations vary from a routine use of $10\,\mu g\,ml^{-1}$ exposed continuously for the duration of culture to a range of concentrations over several logs e.g., 0.1, 1.0, 2.5, and $10.0\,\mu g\,ml^{-1}$ as continuous or 1 h exposure. The drugs are pursued further if the *in vitro* antitumour activity is present at lower two concentrations (Jiang *et al.*, 1983*a,b*; Rozencweig *et al.*, 1984, 1985; Salmon *et al.*, 1981*a,b*; Shoemaker *et al.*, 1984, 1985). The relationship between a given concentration, e.g., $10\,\mu g\,ml^{-1}$, of one drug to that of another drug is completely unknown and can not be defined because the biologic activity of two drugs against human tumours may be quite different at the same concentration. In stead one would prefer to compare antitumour activity of different drugs at similar biologic reference points that correspond to various drug concentrations.

A system that would grade concentrations of various drugs to one or more predetermined biologic reference(s) is possible by utilization of a 'biologic control'. A biologic control that provides various reference points for drugs may help select appropriate *in vitro* concentrations and minimize errors in prediction of activity or inactivity. A biologic control may provide insights into a need for *in vitro* drug bioactivation, drug stability, and would define survival curves. Previously we reported that the breast tumours that were more sensitive *in vitro* than were GM-CFC to myelosuppressive cytotoxic agents, usually responded *in vivo* to the same agents (Hug *et al.*, 1984). We,

Correspondence: J.A. Ajani.
Received 6 March 1986; and in revised form, 2 June 1986.

© The Macmillan Press Ltd. 1986

therefore, examined the utility of human bone marrow granulocyte-macrophage colony forming cells in culture as a biologic reference. It is acknowledged that GM-CFC as a biologic reference would be logical for clinically myelosuppressive agents but less ideal for marrow sparing drugs. However, the myelosuppressive agents do form a majority in the currently available drug armamentarium.

We choose continuous drug exposure over short exposures since the continuous exposure does not significantly alter the activity of cell cycle non-specific agents and yet it is most appropriate for cell cycle specific drugs (Ludwig *et al.*, 1984). There has been an increase in the use of prolonged infusion of cell cycle specific drugs. Clinical correlations have been obtained by utilizing continuous *in vitro* drug exposure (Ajani *et al.*, 1986).

These considerations lead us to conclude that the *in vitro* monitoring of drug activity on normal tissues or malignant cell lines, which exhibit less variability than heterogeneous primary tumours could be of value in: a) understanding the drug concentrations needed to test clinically active drugs, b) defining the *in vitro* biologic activity of investigational agents before screening against human tumours, and c) achieving a ranking system which provides perspective for all drugs. We report *in vitro* activity of 26 chemotherapy drugs, including a number of investigational agents, exposed continuously at various concentrations against the GM-CFC.

### Materials and methods

### *Cell collection and separation*

Bone marrow cells were obtained by aspiration of posterior iliac crest from normal allogeneic donors and from cancer patients with marrow free of tumour infiltration. Aspirates were added to tubes containing 1 ml PBS and 300 units of preservative-free heparin (Fisher Scientific, Houston, Texas). Mononuclear cells were separated by Ficoll-Hypaque (Sigma Chemical Company, St. Louis, Missouri) density-gradient centrifugation ($d = 1.077\,g\,ml^{-1}$).

### *Drug preparation*

All drugs (with the exception of retinoic acid) were diluted with 0.9% NaCl or distilled water, stored in 0.1 ml aliquots at −70°C, and used within one month.

Retinoic acid was dissolved in 95% ethanol at a concentration of 5 mM and stored in the dark at −20°C. Dilutions for addition to the culture system were made in the culture medium containing foetal bovine serum, stored at 4°C, and used within one week. Subdued light conditions were implemented for all retinoic acid work (Findley *et al.*, 1984).

The following commercially available drugs were used: dactinomycin (Merck, Sharp and Dohme, West Point, Pennsylvania), bleomycin sulfate, carmustine, cisplatin, etoposide, mitomycin-C (Bristol Laboratories, Syracuse, New York), cytarabine (Upjohn, Kalamazoo, Michigan), dacarbazine (Miles Pharmaceuticals, West Haven, Connecticut), doxorubicin HCl, 5-fluorouracil (Adria Laboratories, Milan, Italy), all trans retinoic acid (Sigma Chemical Company, St. Louis, Missouri), and vinblastine sulfate and vincristine sulfate (Eli Lilly and Company, Indianapolis, Indiana).

The following investigational drugs were obtained from the National Cancer Institute, Silver Springs, Maryland: caracemide, platinum dichloro-dihydroxybis-(2-propamine)-(OC-6-33) (CHIP), 4-dimethoxydaunorubicin (DMDR), dihydro-5-azacytidine HCl, fludarabine phosphate, ifosfamide, melphalan (i.v. formulation), mitoxantrone HCl, taxol, tiazofurin and nafidimide.

All drug concentrations listed in the experiments are based on the total volume of the agar-medium mixture in the culture dish.

### *Bone marrow culture*

A modified bilayer soft agar system was used for colony formation of GM-CFC (Verma *et al.*, 1980). The 1 ml underlayer consisted of 0.4 ml of 2X alpha minimal essential medium plus 30% foetal bovine serum (MEM; K. C. Biological), 0.2 ml of human placenta conditioned medium, and 0.4 ml of 1.25% Bacto-Agar (Difco, Detroit, Michigan). All cultures were done in 35-mm plastic petri dishes (Corning Glass Works, Corning, New York).

After solidification, the underlayers were overlaid with a 1 ml mixture containing 0.025 ml of graded drug concentrations (0.9% NaCl or distilled water in control plates), 0.4 ml of 2X alpha MEM plus 30% foetal bovine serum, 0.5 ml of 0.75% agar, and 200,000 bone marrow cells in 1X alpha MEM plus 15% foetal bovine serum. In all experiments, triplicate cultures were run for each drug concentration. The cultures were incubated for 8 to 10 days in a humidified atmosphere of 5% $CO_2$, 12% $O_2$, and balanced $N_2$ at 37°C. The GM-CFC (aggregates of 40 or more cells) were scored with an Olympus stereoscopic zoom microscope (Olympus Corporation, New Hyde Park, New York) at 30× magnification. Only cultures with 30 colonies or more (cloning efficiency of 0.02%) were used in the evaluation of drug cytotoxicity. The mean cloning efficiency was 0.06%, with a range of 0.02% to 0.2%.

*Statistical analysis*

Each drug was tested on cells from five different bone marrows. Survival fractions of GM-CFC were calculated as the mean number of colonies in the experimental dishes divided by the mean number of colonies in the control dishes multiplied by 100. Mean and standard deviation were calculated for each drug concentration based on the results of the 5 tests, and the 'best fit' curve was drawn on a semilog scale.

### Results

All 26 chemotherapeutic agents were tested against GM-CFC at at least four different concentrations to generate survival curves. For each drug a pilot experience determined a concentration resulting in some inhibition of GM-CFC. Subsequent concentrations were derived by doubling of the previous one thus resulting in a range of four or more concentrations and yet within a narrow spectrum usually within one log. The highest concentration was up to 4 fold higher than the GM-CFC IC 99. The concentrations resulting in the targeted GM-CFC inhibition were extrapolated from the survival curves. The definition of the targeted GM-CFC inhibitory concentrations (IC), e.g., IC 40, IC 50, IC 78, and IC 90-99, for each drug was considered essential to establish uniform biologic reference points with which all agents could be compared. These values were derived also for the seven investigational agents caracemide, dihydro-5-azacytidine, 4- dimethoxydaunorubicin, ifosfamide, nafidimide, taxol, and tiazofurin.

Based on the shapes of the survival curves generated for 26 drugs, we divided drugs in three main categories: a) Class A drugs – drugs which had initial shoulders on their survival curves but with increasing concentration the curve became steep, b) Class B drugs – drugs which had exponential killing of GM-CFC with initial concentrations but inspite of increasing concentration a plateau was reached, and c) Class C drugs – drugs that demonstrated linear inhibition of the GM-CFC.

Sixteen of 26 (62%) could be classified as Class A drugs producing an initial shoulder. The standard agents in class A category included: dactinomycin, mitoxantrone, vincristine, dacarbazine, 5-fluorouracil, melphalan, and etoposide, and the investigational agents in class A category included: DMDR, taxol, CHIP, ifosfamide, dihydro-5-azacytidine, fludarabine phosphate, nafidimide, spirogermanium, and tiazofurin. The extrapolated GM-CFC IC 40, 50, 78, and 90 to 99 for some of the class A drugs are listed in Table I; these values on the remaining drugs have been published (Umbach *et al.* 1984*a*,*b*; 1985). The parts A and B of Figure 1 depict the survival curves of the representative class A agents e.g., dactinomycin, DMDR, mitoxantrone, taxol, and vincristine. All five drugs achieved GM-CFC IC 99 at a concentration of $<0.0025\,\mu g\,ml^{-1}$.

Four drugs which had GM-CFC survival curves without shoulders but achieved a terminal plateau phase indicating substantial decrease in activity in spite of doubling of the drug concentration. These agents in group B were bleomycin, cytarabine, caracemide, and retinoic acid. The GM-CFC IC 40, 50, 78 and 90 to 98 for these drugs are shown in Table II.

The remaining six drugs constituted group C and had a linear GM-CFC survival curve; meaning a proportionate decrease in the bone marrow cell survival with doubling of the concentration. The drugs in this group included vinblastine, procarbazine (procarbazine being active at concentration above $10\,\mu g\,ml^{-1}$), mitomycin-C, cisplatinum, carmustine and doxorubicin. GM-CFC IC 40, 50, 78 and 90 to 98 for these agents are shown in Table III.

Figure 2 depicts the GM-CFC survival curves generated by dacarbazine, procarbazine, and ifosfamide, all of which require *in vivo* activation for optimal cytotoxic activity. In contrast to the drugs represented in Figure 1, ifosfamide, procarbazine, and dacarbazine resulted in GM-CFC IC 90 at concentration in excess of $10\,\mu g\,ml^{-1}$.

### Discussion

It is difficult to mimic *in vivo* milieu for *in vitro* drug testing. It is difficult also to extrapolate concentration and half life of drugs in the tumour bed with the knowledge of serum concentrations achieved by various dosage and infusion schedules. However, an accurate *in vitro* concentration range would be ideal to avoid erroneous results. A biologic reference in this regard can be utilized to define appropriate *in vitro* concentrations, especially for drugs with unknown human pharmacology.

We used GM-CFC as an *in vitro* biologic reference to define drug concentrations in our assay. The analysis of the survival curves allowed us to classify and understand the dose-response relationship of these drugs. GM-CFC assay may be appropriate for drugs which cause significant myelosuppression clinically. At higher *in vitro* concentrations, the drugs which cause mild or no myelosuppression at maximally tolerated doses, result in inhibition of GM-CFC. Thus the GM-

**Table I** Group A drugs: *In vitro* drug concentration ($\mu g\,ml^{-1}$) for granulocyte-macrophage colony-forming cells.

| *Group A drugs* | *% Inhibitory concentration* 40 | 50 | 78 | 90–98 |
|---|---|---|---|---|
| CHIP | 0.5 | 0.6 | 1.2 | 2.5 |
| Dacarbazine | 60.7 | 71.4 | 114.0 | 250.0 |
| Dactinomycin | 0.0005 | 0.0006 | 0.00096 | 0.00123 |
| 4-Demethoxydaunorubicin | 0.0005 | 0.0007 | 0.0016 | 0.0025 |
| Dihydro-5-azacytidine HCl | 4.4 | 5.3 | 7.4 | 10.0 |
| Etoposide | 0.038 | 0.045 | 0.070 | 0.11 |
| Ifosfamide | 19.9 | 25.1 | 48.9 | 90.0 |
| Mitoxantrone HCl | 0.0006 | 0.00008 | 0.00014 | 0.00025 |
| Nafidimide | 0.083 | 0.094 | 0.13 | 0.2 |
| Taxol | 0.0036 | 0.0040 | 0.0048 | 0.005 |
| Tiazofurin | 0.45 | 0.61 | 1.27 | 2.5 |
| Vincristine sulfate | 0.00076 | 0.0010 | 0.0017 | 0.0025 |

**Figure 1** Granulocyte-macrophage colony-forming cell survival curves for five group A drugs active at concentrations $<0.01\,\mu g\,ml^{-1}$. (a) actinomycin (●); dimethoxydaunorubicin (○); vincristine (△); (b) taxol (L.H. panel) and mitoxantrone (R.H. panel).

CFC assay overestimates their concentrations. Eighteen of 26 drugs achieved GM-CFC IC 99 at concentration $<1\,\mu g\,ml^{-1}$. Only four drugs, caracemide, dihydro-5-azacytidine, retinoic acid, and carmustine, which do not require bioactivation *in vitro*, were inactive at concentration $<1\,\mu g\,ml^{-1}$. This could be contrasted with the concentrations utilized for screening new drugs (Shoemaker *et al.*, 1984; 1985).

We have recently described a human tumour cell assay that uses a tumour matrix for the growth of tumour cells (Ajani *et al.*, 1985; Baker *et al.*, 1986). With *in vitro* sensitivity of human tumours to cytotoxic agents defined as $\geq$50% inhibition of growth, clinically known active agents, e.g., cisplatinum, doxorubicin, 5-fluorouracil, etoposide, mitomycin, and vinblastine had a response rate of 30% or greater when the drugs were tested at concentrations up to GM-CFC IC 90.

Shoemaker *et al.* (1985), for screening of new drugs, have tested a number of agents including actinomycin, spirogermanium, dihydroxyanthracenedione, bleomycin, cisplatinum, 1-Beta-D-arabino furanosylcytosine, vinblastine, carmustine, 5-fluorouracil, vincristine sulphate, taxol, melphalan, procarbazine, and dacarbazine. Procarbazine and dacarbazine were inactive as they resulted in the lowest *in vitro* response rate. However, the myelosuppressive agents such as actinomycin and dihydroxyanthracenedione, two of the three (along with spirogermanium) were the most active drugs but were tested at concentrations 5 to 6 logs higher than GM-CFC IC 50. Spirogermanium was tested at a concentration two logs higher than its GM-CFC IC 50; spirogermanium's dose-limiting toxicity is not myelosuppression but it is neurological, thus, the concentration corresponding to $\geq$GM-CFC IC 50 would be biologically very high. Bleomycin was active also with an *in*

**Table II** Group B drugs: *In vitro* drug concentrations ($\mu g\,ml^{-1}$) for granulocyte-macrophage colony-forming cells

| | *% Inhibitory concentration* | | | |
|---|---|---|---|---|
| *Group B drugs* | *40* | *50* | *78* | *90–98* |
| Bleomycin sulfate | 0.36 | 0.51 | 1.37 | 4.0 |
| Caracemide | 0.57 | 0.93 | 4.72 | 10.0 |
| Cytarabine | 0.0013 | 0.0016 | 0.0025 | 0.0063 |
| Retinoic acid | 2.7 | 3.9 | 9.9 | 19.2 |

**Table III** Group C drugs: *In vitro* drug concentrations ($\mu g\,ml^{-1}$) for granulocyte-macrophage colony-forming cells.

| | *% Inhibitory concentration* | | | |
|---|---|---|---|---|
| *Group C drugs* | *40* | *50* | *78* | *90–98* |
| Carmustine | 1.11 | 1.43 | 2.6 | 5.0 |
| Doxorubicin HCl | 0.0017 | 0.0023 | 0.005 | 0.01 |
| Vinblastine | 0.0003 | 0.0004 | 0.0010 | 0.0025 |
| Cisplatinum | 0.23 | 0.36 | 0.73 | 1.0 |
| Mitomycin-C | 0.0032 | 0.0044 | 0.010 | 0.025 |
| Procarbazine HCl | 48.6 | 60.9 | 109.0 | 200.0 |

**Figure 2** Granulocyte-macrophage colony-forming cell survival curves for three drugs known to require *in vivo* bioactivation. Dacarbazine (●); procarbazine (○); ifosfamide (△).

*vitro* response rate of 33%, however, bleomycin is also nonmyelosuppressive clinically. This is a limitation of the GM-CFC assay. The appropriate *in vitro* concentrations for the mildly myelosuppressive or nonmyelosuppressive agents could be determined accurately by sensitive human tumour cell lines.

•The study by Shoemaker *et al.* (1985) suggests that the *in vitro* activity of clinically known myelosuppressive agents was proportional to the concentration number of logs above the GM-CFC IC 50 (see their Tables 4 and 5). Drugs tested at concentrations closer to GM-CFC IC 50 included cisplatinum, 5-fluorouracil, carmustine, and melphalan resulted in *in vitro* responses consistent with the clinical experience. Vincristine and cytarabine which were both tested at approximately 4 logs above GM-CFC IC 50 had higher *in vitro* activity.

Salmon *et al.* (1984) compared cytotoxic effects of esorubicin with doxorubicin against human tumour samples and normal bone marrow cells and concluded that esorubicin might be more effective and less toxic than doxorubicin. The median

Table IV Comparison of *in vitro* tumor responses and inhibitory concentration (IC-50) for granulocyte-macrophage colony-forming cells (GM-CFC).

| *Drug* | % in vitro[a] *response* | *GM-CFC IC 50 ($\mu g\,ml^{-1}$)* | *10 μg-÷ GM-CFC IC 50* |
|---|---|---|---|
| Actinomycin | 47 | 0.00062 | $1.6\times10^4$ |
| Spirogermanium | 41 | 0.14 | $0.7\times10^2$ |
| Mitoxantrone | 40 | 0.00008 | $1.3\times10^5$ |
| Bleomycin | 33 | 0.51 | $2.0\times10^1$ |
| Cisplatinum | 24 | 0.27 | $3.7\times10^1$ |
| Cytarabine | 22 | 0.0016 | $6.25\times10^3$ |
| Vinblastine | 21 | 0.0004 | $2.5\times10^4$ |
| Carmustine | 21 | 1.43 | 7.0 |
| 5-Fluorouracil | 21 | 0.47 | $2.1\times10^1$ |
| Vincristine | 20 | 0.0010 | $1.0\times10^4$ |
| Taxol | 10 | 0.004 | $2.5\times10^3$ |
| Melphalan | 10 | 0.22 | $4.5\times10^1$ |
| Procarbazine | 8 | 60.9 | 0.16 |
| Decarbazine | 8 | 70.42 | 0.14 |

[a]From Shoemaker *et al.* (1985).

Table V Comparison of *in vitro* tumour responses and inhibitory concentration (IC-50) for granulocyte-macrophage colony-forming cells (GM-CFC).

| *Drugs* | % in vitro[a] *response* | *GM-CFC IC 50 ($\mu g\,ml^{-1}$)* | *10 μg-÷ GM-CFC IC 50* |
|---|---|---|---|
| Actinomycin | 70 | 0.00062 | $1.6\times10^4$ |
| Mitomycin | 69 | 0.0044 | $2.3\times10^3$ |
| Doxorubicin | 65 | 0.0023 | $4.3\times10^3$ |
| Cisplatinum | 54 | 0.27 | $3.7\times10^1$ |
| Bleomycin | 32 | 0.51 | $2.0\times10^1$ |
| Vinblastine | 31 | 0.0004 | $2.5\times10^4$ |
| Carmustine | 23 | 1.43 | 7.0 |
| 5-Fluorouracil | 23 | 0.47 | $2.1\times10^1$ |
| Melphalan | 21 | 0.22 | $4.5\times10^1$ |

[a]From Shoemaker *et al.* (1985), (after establishment of 4 quality controls).

tumour colony IC 50 value for esorubicin was $0.15\,\mu g\,ml^{-1}$ and for doxorubicin was $1.6\,\mu g\,ml^{-1}$, however, the median bone marrow colony IC 50 for esorubicin was $0.002\,\mu g\,ml^{-1}$ only and for doxorubicin was $0.2\,\mu g\,ml^{-1}$. If one were to utilize the GM-CFC assay as a biologic reference then a very low concentration (neary two logs lower than doxorubicin) of esorubicin would be biologically equal to a concentration of doxorubicin and it could be concluded that esorubicin is not superior to doxorubicin.

To screen a new agent, it would be useful to first establish a GM-CFC survival curve then to include the GM-CFC IC range to determine its activity against fresh human tumours. We recommend utility of the malignant cell lines from relatively sensitive tumours as biologic control for clinically nonmyelosuppressive agents.

We conclude that a) the chemotherapeutic agents can be divided into three classes e.g., group A with initial shoulder, group B with terminal plateau, and group C with lineal killing. Such grouping helps in the understanding of dose-dependent cytotoxicity by various agents and provides an insight in their cytotoxic mechanism of action; b) GM-CFC IC 50 for a majority of the drugs is considerably less than

$10\,\mu g\,ml^{-1}$, the range usually selected for screening investigational agents (Shoemaker *et al.*, 1984; 1985), and c) investigational agents showed marked differences in the GM-CFC IC 50 suggesting that arbitrarily chosen concentrations may not give accurate results.

Supported by grants from the National Cancer Institute (grants CA31536 and CA23077), LifeTrac Ltd., Irvine, CA, and the Susan G. Komen Foundation, Dallas, Texas.
G. Spitzer, is a recipient of a scholarship from the Leukemia Society of America.

### References

AJANI, J, BLAAUW, A. & SPITZER, G. (1985). Differential cytotoxic activity of chemotherapy agents on colony forming cells from human tumors and normal bone marrow *in vitro*. *Exp. Hematol*, **13**, 95.

AJANI, J., BAKER, F., SPITZER, G. & 5 others (1986). Adhesive tumor cell culture system (ATCCS): Preliminary results of clinical correlations. *Proc. Am. Assoc. Cancer Res.*, **27**, 411 (# 1631).

ALBERTS, D.S., SALMON, S.E., CHEN, H.-S.G., MOON, T.E., YOUNG, L. & SURWIT, E.A. (1981). Pharmacologic studies of anticancer drugs with the human tumor stem cell assay. *Cancer Chemother. Pharmacol.*, **6**, 253.

BAKER, F., SPITZER, G., AJANI, J. & 9 others (1986). Successful primary monolayer culture of human tumor cells using cell adhesive surface and supplemented medium: colony formation, morphology, karyology, drug, and radiation sensitivity. *Cancer Res.*, **46**, 1263.

FINDLEY, H.W., STEUBER, C.P., RUYMANN, F.B., CULBERT, S. & RAGAB, A.H. (1984). Effect of retinoic acid on the clonal growth of childhood myeloid and lymphoid leukemias: a pediatric oncology group study. *Exp. Hematol.*, **12**, 768.

HUG, B. THAMES, H., BLUMENSCHEIN, G.R., SPITZER, G. & DREWINKO, B. (1984). Normalization of *in vitro* sensitivity testing of human tumor clonogenic cells. *Cancer Res.*, **44**, 923.

JIANG, T.L., LIU, R.H. & SALMON, S.E. (1983*a*). Antitumor activity of didemnin B in the human tumor stem cell assay. *Cancer Chemother. Pharmacol.*, **11**, 1.

JIANG, T.L., SALMON, S.E. & LIU, R.M. (1983*b*). Activity of camptothecin, harringtonin, cantharidin, and curcumae in the human tumor stem cell assay. *Eur. J. Cancer*, **19**, 263.

LUDWIG, R., ALBERTS, D.S., MILLER, T.P. & SALMON, S.E. (1984). Evaluation of anticancer drug schedule dependency using *in vitro* human tumor clonogenic assay. *Cancer Chemother. Pharmacol.*, **12**, 135.

ROZENCWEIG, M., SANDERS, C., ROMBAUT, W. & 4 others (1984). Phase II study of carminomycin in a human tumor cloning assay. *Invest New Drugs*, **2**, 267.

ROZENCWEIG, M., SANDERS, C., ROMBAUT, W., CRESPEICNE, H., KENIS, V. & KLASTERSKY, J. (1985). Phase II study of ametantrone in human tumor cloning assay. *Eur. J. Cancer Clin. Oncol.*, **21**, 195.

SALMON, S.E., LIU, R.H. & CASAZZA, A.M. (1981*a*). Evaluation of new anthracycline analogs with the human tumor stem cell assay. *Cancer Chemother. Pharmacol.*, **6**, 103.

SALMON, S.E., MEYSKENS, JR., F.L., ALBERTS, D.S., SOEHNLEN, B. & YOUNG, L. (1981*b*). New drugs in ovarian cancer and malignant melanoma: *in vitro* phase II screening with the human tumor stem cell assay. *Cancer Treat. Rep.*, **65**, 1.

SALMON, S.E., YOUNG, L., SOEHNLEN, B. & LIU, R. (1984). Antitumor activity of esorubicin in human tumor clonogenic assay with comparison to doxorubicin. *J. Clin. Oncol.*, **2**, 282.

SHOEMAKER, R.H., WOLPERT-DEFILIPPES, M.K. & VENDITTI, J.M. (1984). Potential and drawbacks of the human tumor stem cell assay. *Behring Inst. Mitt.*, **74**, 262.

SHOEMAKER, R.H., WOLPERT-DEFILIPPES, M.K., KERN, D.H. & 8 others (1985). Application of human tumor colony-forming assay to new drug screening. *Cancer Res.*, **45**, 2145.

UMBACH, G., HUG, V., SPITZER, G., THAMES, H. & DREWINKO, B. (1984*a*). Responses of human bone marrow progenitor cells to fluoro-Ara-AMP, homoharringtonine, and elliptinium. *Invest. New Drugs*, **2**, 263.

UMBACH, G., SINGLETARY, S.E., TOMASOVIC, B., SPITZER, G. & DREWINKO, B. (1984*b*). Dose-survival curves of cisplatinum, melphalan and velban in human granulocyte-macrophage progenitor cells. *Int. J. Cell Cloning*, **2**, 335.

UMBACH, G., HUG, V., SPITZER, G. & 4 others (1985). Survival of human bone marrow cells *in vitro* treatment with 12 anticancer drugs and implications for tumor drug sensitivity assays. *J. Cancer Res., Clin. Oncol.*, **109**, 130.

VERMA, D.S., SPITZER, G., BERAN, M., ZANDER, A., MCCREDIE, K.B. & DICKE, K. (1980). Colony stimulating factor: augmentation in human placental conditioned medium. *Exp. Hematol.*, **8**, 917.

VON HOFF, D.D., CASPER, J., BRADLEY, E., SANBACH, J., JONES, D. & MAKUCH, R. (1981). Association between human tumor colony-forming assay results and response of individual patient's tumor to chemotherapy. *Am. J. Med.*, **70**, 1027.